# The role of RObotic surgery in EMergency setting (ROEM): protocol for a multicentre, observational, prospective international study on the use of robotic platform in emergency surgery

**DOI:** 10.1186/s13017-024-00542-x

**Published:** 2024-06-04

**Authors:** Marco Milone, Pietro Anoldo, Nicola de’Angelis, Federico Coccolini, Jim Khan, Yoram Kluger, Massimo Sartelli, Luca Ansaloni, Luca Morelli, Nicola Zanini, Carlo Vallicelli, Gabriele Vigutto, Ernest E. Moore, Walter Biffl, Fausto Catena, ROEM Collaborative Group, Michele Manigrasso, Anna D’Amore, Andrea Pakula, Ibrahim Umar Garzali, Francesk Mulita, Gupta Amit, Maciej Walędziak, Jelle P Ruurda, Antonio Caycedo-Marulanda, Alicia Mangram, Argyrios L Ioannidis, Long R Jiao, Carolina González, Dhaivat Vaishnav, Adeel Ahmed Shamim, Ali F Mallat, Stefano Rossi, Giuseppe Ietto, Pasquale Cianci, Desire Pantalone, Claudia Zaghi, Igor Monsellato, Gianluca Garulli, Vito D’Andrea, Marcello Gasparrini, Micaela Piccoli, Maria Fortuna Offi, Riccardo Memeo, Andrea Coratti, Giuseppe Giuliani, Giampaolo Formisano, Paolo Pietro Bianchi, Benedetto Ielpo, Antonio Giuliani, Louis Perkins, Maximilian Scheiterle, Pietro Coletta, Irnerio Muttillo, Jerzy Draus, Jacopo Andreuccetti, Georgios-Ioannis Verras, Michał Wiłkojć, Richard van Hillegersberg, Alexis Sanchez, Konstantinos Konstantinidis, Fabio Ausania, Biagio Picardi, Ivana Conversano, Gherardo Maltinti, Giulia Costantini, Mattia Portinari, Maria Irene Bellini, Federica Cosmi, Sofia Esposito, Rosalinda Filippo, Fabio Vistoli, Maricn Zawadzki, Barbara J Broome, Michael Konstantinidis, Caterina Puccioni, Enrico Restini, Anna Maria Di Bella, Gianmarco Palini, Maria Ludovica Costanzo, Matteo Gasparrini, Alice Francescato, Antonella Delvecchio, Barbara Mullineris, Pierfrancesco Lapolla, Andrea Mingoli, Gioia Brachini, Simone Guadagni, Francesco Matarazzo

**Affiliations:** 1 Department of Clinical Medicine and Surgery, Federico II University, Via Pansini 5, 80131 Naples, Italy; 2 Unit of Colorectal and Digestive Surgery, DIGEST Department, Beaujon University Hospital, AP-HP, University of Paris Cité, Paris, France; 3 General Surgery Unit, University of Pisa, Pisa, Italy; 4 University of Portsmouth, Portsmouth Hospitals University NHS Trust, Portsmouth, UK; 5 Department of General Surgery, Rambam Health Care Campus, Haifa, Israel; 6 Department of Surgery, Macerata Hospital, Macerata, Italy; 7 Department of Surgery, Fondazione IRCCS Policlinico San Matteo, University of Pavia, Pavia, Italy; 8 General and Emergency Surgery Department, Bufalini Hospital, Cesena, Italy; 9 Ernest E Moore Shock Trauma Center at Denver Health, University of Colorado, Denver, CO USA; 10 Division of Trauma/Acute Care Surgery, Scripps Clinic Medical Group, La Jolla, CA USA

**Keywords:** Emergency surgery, Robotic surgery, ROEM study

## Abstract

**Background:**

Robotic surgery has gained widespread acceptance in elective interventions, yet its role in emergency procedures remains underexplored. While the 2021 WSES position paper discussed limited studies on the application of robotics in emergency general surgery, it recommended strict patient selection, adequate training, and improved platform accessibility. This prospective study aims to define the role of robotic surgery in emergency settings, evaluating intraoperative and postoperative outcomes and assessing its feasibility and safety.

**Methods:**

The ROEM study is an observational, prospective, multicentre, international analysis of clinically stable adult patients undergoing robotic surgery for emergency treatment of acute pathologies including diverticulitis, cholecystitis, and obstructed hernias. Data collection includes patient demographics and intervention details. Furthermore, data relating to the operating theatre team and the surgical instruments used will be collected in order to conduct a cost analysis. The study plans to enrol at least 500 patients from 50 participating centres, with each centre having a local lead and collaborators. All data will be collected and stored online through a secure server running the Research Electronic Data Capture (REDCap) web application. Ethical considerations and data governance will be paramount, requiring local ethical committee approvals from participating centres.

**Discussion:**

Current literature and expert consensus suggest the feasibility of robotic surgery in emergencies with proper support. However, challenges include staff training, scheduling conflicts with elective surgeries, and increased costs. The ROEM study seeks to contribute valuable data on the safety, feasibility, and cost-effectiveness of robotic surgery in emergency settings, focusing on specific pathologies. Previous studies on cholecystitis, abdominal hernias, and diverticulitis provide insights into the benefits and challenges of robotic approaches. It is necessary to identify patient populations that benefit most from robotic emergency surgery to optimize outcomes and justify costs.

## Introduction

Robotic surgery has progressively gained acceptance in several surgical fields, being routinely used for elective interventions [1–3]. The issue regarding the role of robotic surgery for emergency procedures remains open. Few studies have been published regarding the applications of robotics for emergency general surgery procedures; they were reviewed and discussed in the 2021 WSES position paper [4]. Studies on colorectal surgery, hiatal hernia surgery, bariatric surgery, gallbladder surgery, and abdominal wall surgery were included and statements proposed. The experts recommended a strict patient selection, an adequate training of the operating surgical team and an improvement of the accessibility of the robotic platforms. We propose this prospective study to better define the application of robotic surgery in an emergency setting, evaluating the intraoperative and postoperative outcomes, trying to understand the role of the robotic platform in the management of emergency situations.

## Methods

### Study objectives

The primary aim of the ROEM study is to evaluate safety and feasibility of robotic surgery in patients requiring an emergency treatment for acute surgical pathologies, examining intraoperative and postoperative outcomes, assessing the role of robotic platform in emergency setting. The secondary aim is to conduct a cost analysis to understand whether high costs actually represent a limit for robotic surgery.

### Study design

ROEM is an observational, prospective, multicentre, international study. Data of clinically stable patients who underwent robotic surgery in emergency setting will be prospectively analysed. The pathologies that will mainly be taken into consideration will be acute diverticulitis, acute cholecystitis and obstructed hernias. The Hinchey classification will be used to describe the degree of acute diverticulitis [5], and the 2018 Tokyo guidelines will be used to describe the degree of acute cholecystitis [6]. Patients with other surgical pathologies may also be enrolled in the study as long as they are treated in robotic surgery in emergency setting. Data relating to the operating theatre team and the surgical instruments used will be collected in order to conduct a cost analysis. The variables under study are listed in Table 1.

**Table 1:** Variables under study

*Demographics*
Patient sex
Patient age
BMI
Age-adjusted Charlson Comorbidity Index (aCCI)
Previous surgery
*Disease requiring emergency surgery*
Diagnosis (acute diverticulitis, acute cholecystitis, obstructed hernia, other)
Hinchey grading
Tokyo grading
Site of hernia
*Operative data*
Amount of robotic surgeries performed in the institution (*per year*)
Surgeon expertise (*robotic surgeries performed per year*)
Robot model
Type of procedure
Time of intervention (what time of day was the surgery performed?)
Operative time
Intraoperative complications
Conversion
Reason of conversion
Drain
*Recovery*
Length of stay in ICU (*stay in intensive care unit*)
Clavien-Dindo
Type of complication
Treatment of complication
Death
Time to first flatus
Time to first mobilization
Time to first oral feeding
Length of stay
90-days mortality
90-days readmission
*Theatre staff and consumable equipment (amount)*
Ordinary consultant surgeons
Dedicated consultant surgeons
Ordinary non-consultant surgical assistants
Dedicated non-consultant surgical assistants
Ordinary anesthetic consultants
Dedicated anesthetic consultants
Ordinary non-consultant anesthetists
Dedicated non-consultant anesthetists
Ordinary theatre nurse scrubber
Dedicated theatre nurse scrubber
Ordinary theatre nurse circulating
Dedicated theatre nurse circulating
Ordinary portering staff
Dedicated portering staff
Maryland bipolar forceps
Fenestrated bipolar forceps
Permanent cautery hook
Cadiere forceps
Hot shears
Prograsp forceps
Vessel sealer
Harmonic ace
Staplers
Stapler reloads
Sutures
Drains
Hemostatic consumables
Diathermy consumables
Scrub suits
Dressings
Drapes

### Study setting and sample size

Any centre performing robotic surgery for emergency procedure will be eligible to participate. Each centre will have a local lead and up to two collaborators; data can be provided for the entire study period, the expected duration of which is at least one year.

Since it is not routine to use the robotic platform in an emergency setting, there is no data in the literature regarding an estimate of the number of patient subject to these procedures. Assuming that at least one emergency robotic intervention is carried out per month and that 50 centres participate, we estimate a minimum sample size of 500 patients.

### Eligibility criteria

The following criteria must be satisfied for patient inclusion in the study:

- adult patients (18 years or above);
- clinically stable patients with disease requiring emergency surgical treatment;
- intervention performed in robotic surgery;
- capability of giving valid informed consent.

Patients who fulfil any of the following criteria will be excluded:

- patients under 18 years of age;
- intervention performed in open or laparoscopic surgery;
- elective surgery;
- clinically unstable patients;
- inability of giving valid informed consent.

### Data collection and management

All data will be collected and stored online through a secure server running the Research Electronic Data Capture (REDCap) web application. REDCap allows collaborators to enter and store data in a secure system. Collaborators will be given secure REDCap project server login details, allowing secure data storage on the REDCap database. No patient-identifiable information will be uploaded, and anonymized data will be pooled and analysed, with no surgeon- or centre-specific comparisons performed.

### Statistical analysis

Data will be expressed as median and interquartile range (IQR) and number and relative percentage. Normal distribution of continuous variables will be assess with the Kolmogorov–Smirnov test. Continuous variables will be analysed using the student *t*-test or Mann–Whitney test and categorical variables using Fisher exact test or Chi-Square test as appropriate. Significant variables (*p* < 0.05) at univariate and well-known variables affecting outcomes will be used to run the matching. All statistics will be 2-tailed and statistical significance will be accepted when *p* < 0.05. All statistical analyses will be performed using IBM SPSS Statistics 27.

## Discussion

As state in the 2021 WSES position paper after a careful review of the literature and a consensus of experts [4], the use of robotic surgery in emergency setting is feasible if properly supported by surgical staff and equipment. They selected ten papers focusing on emergencies in colorectal, hiatal hernia, gallbladder, bariatric and abdominal wall surgery.

On the same page, they also point out how the use of robotic surgery, especially with the aid of telemedicine, will be useful in surgical training for a minimally invasive surgery curriculum. Considering the progression on the learning curve, WSES in 2022 [7] publishes a position paper on how a proper training curriculum in minimally invasive surgery in emergency setting should be created. Most of the studies considered are about laparoscopic surgery, nonetheless they included a study on single port robotic cholecystectomies and two studies on strangulated inguinal hernias. All the studies show an increased spectrum of difficulty in emergency setting, so they propose to focus on robotic emergency surgeries after completing the elective learning curve.

Aside from the need of trained staff, the use of robotic surgery in emergencies should not interfere with elective surgery [4]. As already pointed out in 2021 by Sudan [8], the increased amount of possible elective cases and the number of surgeons trained in robotic surgery force hospitals to consider new schedules of robotic surgery, like after hours or on the weekends. They present two cases of complicated bariatric surgery both performed with the robot with excellent results, although they notice how fundamental is having an available trained staff and a compliant anesthesiologist.

Focusing on how robotic surgery in emergency setting is feasible, Reinisch [9] publish a systematic review of the literature considering 52 papers. They divide emergencies in appendectomy, cholecystectomy, abdominal wall surgery and other procedures. The review confirms what already shown in the WSES paper [4]: there is not an increased amount of complications and robotic surgery is a valid option in emergency setting. Although they report the impossibility to publish a proper meta-analysis due to the lack of enough published papers on robotic surgery in emergency setting, the data suggest an increased operative time than laparoscopy and an increased cost per operation without considering the initial cost of purchasing the robotic equipment.

In 2023 Grimsley [10] publish a multicentric retrospective study on the difference between the cost of laparoscopic emergency surgery and robotic emergency surgery. Between 2018 and 2020, they analized all the data linked to the operative and post-operative costs for all emergency general surgery operations both robotic and laparoscopic. Similar to Reinisch [9], they find an increased hospital cost performing robotic emergency surgery than laparoscopic without any evident benefit for patients' outcome, so they propose to further studies to identify the proper patients' population to receive the most benefit compared to a similar cost than the same laparoscopic procedure.

In this study, we will focus mainly on three most common disease treated with emergency surgery, such as diverticulitis, cholecystitis and hernias. Nevertheless, we will consider also all the other less common emergency surgical procedure for other surgical diseases, but they will be categorized together as “other disease”.

Considering the cholecystitis, laparoscopic cholecystectomy is now the standard approach, but there are several studies in literature which explore the possibility of using robotic surgery to perform better especially in case of perforated or gangrenous cholecystitis. Milone [11] report three cases of emergency robotic cholecystectomy, two were empyematous and one gangrenous. They reported a slight increase in operative time with a discharge within 24 h from the procedure and no complications afterward. As literature shows, they confirm robotic cholecystectomy is feasible and safe with better outcome in gangrenous cholecystitis than standard laparoscopic cholecystectomy.

The second disease is abdominal hernias which can be approached both laparoscopic or open in emergency setting. Kudsi [12] offer a comparison between the outcome of open approach and robotic approach in emergency setting. They selected 43 patients who underwent the open approach and 35 who underwent the robotic one. As supposed, they notice an increased operative time and fewer complications, such as surgical site infections. However, both the length of stay or the recurrence differed in both groups.

Last one is diverticulitis that, as before, can be approached both laparoscopic or open in emergency setting due to surgeon expertise and technical difficulty. Curfman [13] offer a retrospective multicentric review comparing the open, laparoscopic and robotic approach of emergent colorectal resection for acute diverticulitis. They consider data from 262 facilities from 2018 to 2021 and they're comparing open with robotic and laparoscopic with robotic approach. Robotic surgery shows an increased operative time than both laparoscopic and open approach. At the same time, robotic surgery offers a decreased ICU admission rates and anastomotic leak rates than open approach and reduced anastomotic leak rates and conversion rates than laparoscopic approach.

At last, we regroup all other robotic emergency procedures together in order to consider also rarer procedures in emergency setting that could benefit a robotic approach. For example, Robinson [14] focuses on gastrojejunal ulcers in patients with Roux-en-Y gastric bypass and compares laparoscopic and robotic approach obtaining an improved start time and an increased cost in robotic approach, but no other significant results. Aside from acute diverticulitis and considered the increasing interest in elective robotic colorectal surgery, Maertens [15] offer a retrospective case series on ten emergent robotic colorectal surgeries getting a R0 associated with a proper lymph node dissection and no major complication or 30-day mortality. Another case of oncologic emergency surgery is reported by Conticchio [16] with a ruptured hepatocellular carcinoma treated with a robotic approach in a stable patient after trans arterial embolization failure. Last case series reported is by Ceccarelli [17] about five patients affected by strangulated hiatal hernias treated with laparoscopic or robotic approach. After performing the diagnosis with CT scan, three patients underwent robotic approach and two laparoscopic approach without complications o recurrence. Although there are no recommendations in current literature, robotic surgery appear to be feasible in stable patients in this emergency setting.

## Data Availability

The datasets used and/or analysed during the current study will be available from the corresponding author on reasonable request.

## References

[1] Jung M (2015). Robotic generals surgery: current practice, evidence, and perspective. Langenbecks Arch Surg.

[2] Felder SI (2018). Robotic gastrointestinal surgery. Curr Probl Surg.

[3] Liu R (2019). International consensus statement on robotic pancreatic surgery. Hepatobiliary Surg Nutr.

[4] De Angelis N (2022). Robotic surgery in emergency setting: 2021 WSES position paper. World J Emerg Surg.

[5] Hinchey EJ (1978). Treatment of perforated diverticular disease of the colon. Adv Surg.

[6] Okamoto K (2018). Tokyo Guidelines 2018: flowchart for the management of acute cholecystitis[published correction appears in J Hepatobiliary Pancreat Sci. 2019 Nov;26(11):534]. J Hepatobiliary Pancreat Sci..

[7] De Angelis N (2023). Training curriculum in minimally invasive emergency digestive surgery: 2022 WSES position paper. World J Emerg Surg.

[8] Sudan R (2012). Emergency and weekend robotic surgery are feasible. J Robotic Surg.

[9] Reinisch A (2023). Robotic operations in urgent general surgery: a systematic review. J Robot Surg.

[10] Grimsley EA (2023). Patient outcomes and cost in robotic emergency general surgery. J Robot Surg.

[11] Milone M (2019). Robotic cholecystectomy for acute cholecystitis Three case reports. Medicine.

[12] Kudsi OY (2021). Comparison of midterm outcomes between open and robotic emergent ventral hernia repair. Surg Innov.

[13] Curfamn KR (2023). Robotic colorectal surgery in the emergent diverticulitis setting: is it safe? A review of large national database. Int J Colorectal Dis.

[14] Robinson TD (2022). Emergent robotic versus laparoscopic surgery for perforated gastrojejunal ulcers: a retrospective cohort study of 44 patients. Surg Endosc.

[15] Maertens V (2022). Emergency robotic colorectal surgery during the COVID-19 pandemic: a retrospective case series study. Laparosc Endosc Robotic Surg.

[16] Conticchio M (2023). Robotic emergency liver resection of ruptured hepatocellular carcinoma. Int J Med Robot.

[17] Ceccarelli G (2020). Minimally invasive laparoscopic and robotassisted emergency treatment of strangulated giant hiatal hernias: report of five cases and literature review. World J Emerg Surg.

